# Medical Education of Women

**Published:** 1869-12

**Authors:** 


### Editorial Department.

### Medical Education of Women.

Many of our exchanges indulge in expressions of opinion upon the medical education of women and their admission to our schools and colleges; and it is quite proper that, as a profession, we define our position, and make known the platform upon which we stand in this important question of the propriety of admitting woman to medical colleges, and their natural qualifications as members of the medical profession. In the first place it should be very distinctly stated, that all *gentlemen* are in favor of every thing which can promote the real prosperity, success and happiness of the female sex; and that the question is fully settled when we have determined the influence which it will have upon it. It will be said that this question of the eligibility of women to the office of physicians and surgeons is already settled, that they now occupy these responsible places with credit to themselves and honor to the profession; but this question is still open for discussion, and though some most estimable ladies have honored the degree of Doctor in Medicine, it is still not settled that the degree of Doctor in Medicine is an honor to any real lady. If women, after due reflection and enlightened reasoning upon the subject, yet desire to be physicians, and to practice the art of medicine, it seems as though it was one of the "inalienable rights," and that they should certainly be allowed to do for themselves what they choose in this respect. Woman has a right to enter the clerical profession and minister at the altar, or the legal profession and plead at the bar; she has a right—natural right—to enter into any political or commercial enterprise she may desire; in a word, she has a right to seek her own interest and happiness in her own way, and, so far as the other sex are concerned, they are under obligation to aid and encourage her in every effort which is calculated to promote her welfare. They are excluded from clinical teaching on account of "impropriety of time and place;" but, if they are to practice the profession, they are to forget sexual differences, as physicians in the presence of pain and disease, forget them, are to know nothing but disease and its modes of cure; this is to be the very first step in the process of education. If a woman is educated as a physician, and practices the profession, she can never more be looked upon and protected as that chaste, sensitive, modest, innocent and confiding being which men so naturally honor, cherish and love. She must be supposed to be familiar with all the conditions of humanity, male and female; and in her thoughts, in her knowledge, and in her business, to have so changed as to rob her of all the qualities which have thus far been her strength, her protection and her charm. If women are to be physicians, from no place where is disease and pain can they be excluded on the flimsy plea of "impropriety," for she must be educated to meet these emergencies; if they are to be surgeons, wherever surgical operations are made she must be admitted, for surgeons must be familiar with all surgical regions, and must be prepared at all times, without delay or embarrassment, to institute surgical relief, if called upon. If educated to the profession, it must be done thoroughly; for, certainly we will not consent to making quacks and charlatans of them—the most contemptible, low and dishonest of all thieves and liars. But these are not the considerations which would most influence this question. The inquiry should first be made, What effect will the elective franchise, the professions, and unrestricted and equal property rights, if bestowed as fully on women as men, have upon the mutual relations of dependence and protection now felt by men and enjoyed by women? "Women's rights!" they are many, and may God protect them. If I were to plan, with malicious hate and evil intention, the greatest curse I could conceive for women; if I would estrange them from the protection of heaven, and make them, as far as possible, loathsome and disgusting to men, I would favor the reform, or advancement, as they please to term it, which some of the male-female sex have proposed for them. I would make Ministers, and Lawyers, and Doctors, and Politicians of them. I would have them hold their own estates, independent of their husbands, in their own names; in a word, I would make, as near as possible, men of them, and encourage them to set up life "on their own hook." It is said, by the silly and simple, that women are denied equality with men, are excluded from their opportunities of education and culture, and, of course, cannot equal them in attainment; and that if we but open to them our colleges and hospitals, they will become dangerous rivals. They are assistants, not rivals; in some respects they are the superiors, and can excel. Woman is a welcome attendant in the sick room; whenever excluded, there is gloom and sadness, and privation. With her is cheerfulness and comfort, cleanliness and hope. Truly, she has her sphere; and, in her place, she is vastly superior to man. Indeed, men of even considerable genius find it very embarrassing and inconvenient to do the duty and fill acceptably the places appropriate for women. Nothing a true woman shall desire for herself will ever be long denied her. She is next to the Angels in the Divine favor, and first in the love and veneration of men. Whatever she persistently asks, she is sure to gain, and there is no use to oppose or attempt to discourage. If women desire to become members of the learned professions; if they conclude that they can adorn these places, [adorn is the word: they have naturally a desire to be ornamental,] then there can be no middle ground, they must be admitted. We can see no objection so far as men are concerned; and if we could, we would waive it in favor of women.

The students in Philadelphia, who cheered the young ladies from the clinic, were, perhaps, thoughtless and rude, but there was no evil intention. Those young men believed that the exhibition was improper for young ladies, and that their feelings of delicacy would be outraged if they remained; they had not considered that they were about to become physicians, and were expected to have no sensibility common to the sex. If women earnestly desire medical education, it cannot be denied them—possibly a more extended trial of its general feasibility and appropriateness may convince better than argument or persuasion.

